# Mature miR-99a Upregulation in the Amniotic Fluid Samples from Female Fetus Down Syndrome Pregnancies: A Pilot Study

**DOI:** 10.3390/medicina55110728

**Published:** 2019-11-07

**Authors:** Anda-Cornelia Vizitiu, Danae Stambouli, Anca-Gabriela Pavel, Maria-Cezara Muresan, Diana Maria Anastasiu, Cristina Bejinar, Anda Alexa, Catalin Marian, Ioan Ovidiu Sirbu, Laurentiu Sima

**Affiliations:** 1 Doctoral School, Victor Babes University of Medicine and Pharmacy Timisoara, Eftimie Murgu Nr. 2, Timisoara 300041, Romania; anda.vizitiu@umft.ro; 2 CytoGenomic Medical Laboratory, Calea Floreasca Nr. 35, Sector 1, Bucharest 014451, Romania; cytogenomic@cytogenomic.ro (D.S.); pavel@cytogenomic.ro (A.-G.P.); 3 Obstetrics and Gynecology Department, Victor Babes University of Medicine and Pharmacy, Eftimie Murgu Nr. 2, Timisoara 300041, Romaniaanastasiu.diana@gmail.com (D.M.A.); 4 Biochemistry Department, Victor Babes University of Medicine and Pharmacy, Eftimie Murgu Nr. 2, Timisoara 300041, Romania; tynabej@yahoo.com (C.B.); anda_creanga@yahoo.com (A.A.); cmarian@umft.ro (C.M.); 5 Surgical Semiology Department, Victor Babes University of Medicine and Pharmacy, Eftimie Murgu Nr. 2, Timisoara 300041, Romania; lica_sima@yahoo.com

**Keywords:** Down syndrome, amniotic fluid, pregnancy, microRNA, cardiac defects

## Abstract

*Background and Objective:* Although Down syndrome is the most frequent aneuploidy, its pathogenic molecular mechanisms are not yet fully understood. The aim of our study is to quantify—by qRT-PCR—the expression levels of both the mature forms and the pri-miRNAs of the microRNAs resident on chromosome 21 (miR(21)) in the amniotic fluid samples from Down syndrome singleton pregnancies and to estimate the impact of the differentially expressed microRNAs on Down syndrome fetal heart and amniocytes transcriptomes. *Materials and methods:* We collected amniotic fluid samples harvested by trained obstetricians as part of the second trimester screening/diagnostic procedure for aneuploidies to assess the trisomy 21 status by QF-PCR and karyotyping. Next, we evaluated—by Taqman qRT-PCR—the expression levels of both the mature forms and the pri-miRNA precursors of the microRNAs resident on chromosome 21 in amniotic fluid samples from singleton Down syndrome and euploid pregnancies. Further, we combined miRWalk 3.0 microRNA target prediction with GEO DataSets analysis to estimate the impact of hsa-miR-99a abnormal expression on Down syndrome heart and amniocytes transcriptome. *Results:* We found a statistically significant up-regulation of the mature form of miR-99a, but not pri-miR-99a, in the amniotic fluid samples from Down syndrome pregnancies with female fetuses. GATHER functional enrichment analysis of miRWalk3.0-predicted targets from Down syndrome amniocytes and fetal hearts transcriptome GEODataSets outlined both focal adhesion and cytokine–cytokine receptor interaction signaling as novel signaling pathways impacted by miR-99a and associated with cardiac defects in female Down syndrome patients. *Conclusions:* The significant overexpression of miR-99a, but not pri-miR-99a, points towards an alteration of the post-transcriptional mechanisms of hsa-miR-99a maturation and/or stability in the female trisomic milieu, with a potential impact on signaling pathways important for proper development of the heart.

## 1. Introduction

Down syndrome (DS) represents the most common aneuploidy in humans, with an overall incidence of 1/650–1000 live births and a pregnancy loss rate of only 30% between 12–40 weeks of gestation. Prenatal molecular diagnosis of DS relies mostly on invasive procedures like chorionic villous (CV) and amniotic fluid (AF) sampling, which bear a risk (1%–2%) of pregnancy loss [1,2].

DS associates a stunningly diverse array of health problems, from cognitive impairment, autoimmune disorders and Alzheimer disease, to cardiac defects [3]. The current understanding of the pathogenic mechanisms underlying DS phenotypes gravitates around two non-exclusive main theories: the “gene dosage”, postulating an imbalance in the expression of genes mapped to chromosome 21, and the “developmental instability”, postulating non-specific alterations of chromosomal imbalances [4,5].

MicroRNAs are short (20–25 nucleotides) non-coding RNA molecules involved in post-transcriptional regulation of gene expression through direct interaction with target mRNAs. Throughout DS pregnancy, distinct fetal, placental, amniotic fluid and maternal plasma microRNA signatures have been associated with physiological and pathological aspects of both fetus and infant development [6,7,8,9,10,11,12,13,14,15].

Only five of the microRNAs listed by miRBase as mapped to chromosome 21 (miR(21)) have been experimentally confirmed: hsa-miR-99a (miR-99a), hsa-let-7c (let-7c), hsa-miR-125b-2 (miR-125b), hsa-miR-155 (miR-155) and hsa-miR-802 (miR-802) [16]. The first three belong to a cluster located within the C21orf34, miR-802 is located inside RUNX1, while miR-155 is located within MIR155HG (MIR155 host gene) as part of BIC (B cell integration) cluster. Bioinformatics analyses of miR(21) predict over 2000 putative targets, which suggest that if deregulated, miR(21) might play an important role in DS pathogenesis [17].

Cardiac-specific overexpression of miR(21) in DS hearts might play a distinct post-transcriptionally regulatory role, part of the overall picture of imbalanced microRNA expression contributing to gene dosage effects in DS individuals. Furthermore, the miR-99 family of evolutionarily conserved microRNAs is known for its role in cardiomyogenesis, heart regeneration, cardiac hypertrophy, and post-infarction cardiac remodeling.

Here, we analyzed the expression of miR(21) in amniotic cell samples from DS and euploid singleton pregnancies, and evaluated the impact of the differentially expressed microRNAs on DS heart and amniocyte transcriptomes. To the best of our knowledge, this is the first report pointing towards a post-transcriptional alteration of microRNA processing in the DS cells.

## 2. Materials and Methods

### 2.1. Study Subjects

In this study, we included patients with high-risk DS pregnancies after prenatal screening and referred for the DS prenatal diagnosis by amniocentesis to the Premiere Hospital, County Hospital Timisoara, Romania and the Cytogenomic Medical Laboratory. All the participants provided a written consent for sample collection and tissue molecular analysis. Our study was approved by the Ethical Committee of the Municipal County Hospital (#73/20.12.2014) and was conducted according to the principles of the Helsinki Declaration.

### 2.2. Sample Collection and Down Syndrome Diagnostic

The transabdominal sampling of amniotic fluid using 20-gauge needles was performed by trained obstetricians as part of the second-trimester screening/diagnostic procedure for aneuploidies. Half of the AF samples was used for rapid detection of common autosomal aneuploidies by quantitative fluorescent PCR (QF-PCR) using the ChromoQuant kit (Cybergene, Solna, Sweden). The other half was further split into three independent flasks and cultured in Chang Medium supplemented with L-glutamine (Irvine Scientific, Santa Ana, CA, USA). Two cultures were used for karyotyping, while the cells from the third culture were collected, centrifuged for 10 min at 1000 rpm and 4 °C and then stored in RNAlater at −80 °C until further use.

### 2.3. Gene Expression Analysis

We used TaqMan^®^ MicroRNA Cells-to-CT™ Kit and TaqMan^®^ Gene Expression Cells-to-CT™ Kit (Thermo Fisher, Waltham, MA, USA) to quantify microRNA and pri-miRNA, respectively, in the AF lysates, according to the manufacturer’s instructions. Real-Time PCR was performed in 96-well plates on a 7900HT Fast Real-Time PCR System using inventoried TaqMan microRNA and pri-miRNA Assays (Thermo Fisher, Waltham, MA, USA). All qRT-PCR reactions were performed in triplicate and normalized to RNU49 (microRNA quantification) and GAPDH (pri-miRNA quantification).

### 2.4. Statistical Analyses

Demographic data are presented as mean ± standard deviation and analyzed using the heteroscedastic, two-tailed Student *T* test with Welch’s correction; sex ratios were analyzed using the two-tailed *Z*-test. Normalized values of microRNAs and pri-microRNAs were statistically analyzed using a heteroscedastic, two-tails Student-*T* test with Welch’s correction for normally distributed data (Kolmogorov–Smirnov test, alpha = 0.05) and Mann–Whitney test for abnormally distributed data. All statistics calculations were performed using Prism 8 for MacOS, version 8.2.0 (GraphPad Software, San Diego, CA, USA).

### 2.5. Target Predictions and Bioinformatics Analysis

We performed microRNA target predictions using the miRWalk3.0 TarPmiR machine learning algorithm and cross-referenced to the set of genes deregulated in DS hearts and DS amniocytes (GSE16176, GSE1397; GSE1789) [18,19,20]. KEGG pathways analysis was performed using the GATHER network-inferred, homologues-included algorithm (Bayes factor ≥ 10), (http://changlab.uth.tmc.edu/gather/gather.py) [21].

## 3. Results

We used qRT-PCR to evaluate the expression of miR(21) in 39 AF cultured cell samples from DS (*n* = 17) and euploid (*n* = 22) pregnancies. All the patients included in the study presented high-risk DS pregnancies after the prenatal screening and consented to the invasive prenatal diagnosis of DS by amniocentesis. Of note, the difference between the gestational age of DS vs. euploid pregnancies is statistically significant due to a unique outlier, in the absence of which, this difference becomes irrelevant. The characteristics of the AF samples are outlined in Table 1.

**Figure 1:**
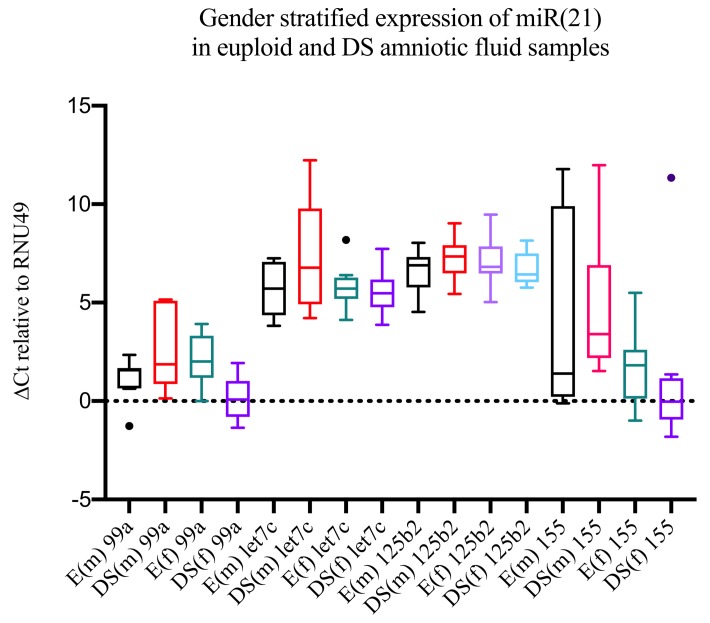
Box-and-whiskers Tukey plot representation of gender-stratified (males, m; females, f) microRNA expression in Down syndrome (DS) and euploid (E) samples.

miR-802 was excluded from our analysis because over 70% of (both the DS and euploid) samples were scored *undetermined* (threshold C_t_ = 40). With the exception of miR-99a in pregnancies with a female fetus (FC = 3.84; *p* = 0.0005), none of the microRNAs investigated were differentially expressed in DS vs. euploid samples (Figure 1, Table 2).

Cell- and gene-specific transcriptional modulation has been observed in cell culture and could account for the mature microRNA quantification results [22,23,24]. In order to assess the possible transcriptional activation of the microRNA gene, we quantified the corresponding pri-miR-99a levels and found no statistically significant changes (Table 2). This suggests that the up-regulation of miR-99a involves post-transcriptional mechanisms leading to an augmented stability/decreased degradation rate of the mature microRNA.

Next, we compared the microRNA expression levels between male and female in both DS and euploid samples and found statistically significant differences in miR-99a (FC = 5.12; *p* = 0.040) and miR-155 (FC = 0.08; *p* = 0.0031) DS samples (Table 3). Of note, only two results withstood the Bonferroni correction: the miR-99a change in female samples (DS vs. euploid, with an adjusted *p* = 0.0025) and the miR-155 change in DS samples (males vs. females, with an adjusted *p* = 0.015).

Several lines of evidence collectively indicate a possible link between miR-99a and cardiac defects in female trisomic fetuses. First, together with miR-155 and let-7c, miR-99a is overexpressed in the DS fetal hearts associating congenital heart defects (CHD) [25]. Second, miR-99a is involved early in cardiomyogenesis, through SMARCA5 modulation of Nodal/Smad2 signaling [26]. Furthermore, elevated miR-99a maternal plasma levels have been found to be associated with fetal congenital heart defects, and, given the rapid, bidirectional communication between the fetus and the AF through the amnion, this indicates the fetal heart as possible source of the plasma miR-99a [27,28]. Last, but not least, there is a well-known prevalence of cardiac septal defects (atrio-ventricular in particular) in female DS patients, the ethiopathogenic molecular basis of which is not currently understood [29,30,31].

In order to reveal the biological significance of miR-99a up-regulation in AF samples, we used miRWalk 3.0 to compile the list of predicted (3’-UTR, CDS and 5’-UTR) miR-99a targets (2134 unique entries). Next, we cross-referenced this list against the set of genes deregulated in DS fetal amniocytes (GSE16176) and fetal hearts (GSE1397, GSE1789) retrieved after Geo2R analysis (*p* < 0.05) and obtained two sets of 187 and 208 unique DS-related genes, respectively (Table S1). Further functional enrichment analysis (adjusted *p* < 0.05, Bayes factor ≥ 10) using the GATHER algorithm retrieved two significantly enriched KEGG pathways potentially targeted by miR-99a in both amniocytes and heart cells (Table 4): Focal adhesion and cytokine–cytokine receptor interaction.

Our analysis of miR(21) expression in amniotic cell samples from DS and euploid singleton pregnancies shows an increased expression level of mature miR-99a alone (but not pri-miR) in AF cells sampled from female fetus DS pregnancies, which may significantly impact signaling pathways involved in heart morphogenesis.

## 4. Discussion

To the best of our knowledge, this is the first communication of data indicating a possible alteration of post-transcriptional mechanisms of microRNA maturation/decay in DS tissues. Furthermore, in line with previous data, our analysis shows a significant enrichment in mature miR-99a (but not in pri-miR precursor) in AF samples from DS female fetus pregnancies, raising the question whether it could serve as biomarker for CHD [25].

Heart development involves multiple, partially overlapping steps controlled by an evolutionary conserved network of signaling pathways and transcriptional regulators, among which microRNAs in general (and miR-99a in particular) play important roles [32]. miR-99a is a member of the miR-99 family (miR-99a, miR-99b, and miR-100) of evolutionary conserved microRNAs, known for their role in cardiomyogenesis, heart regeneration, cardiac hypertrophy and post-infarction cardiac remodeling [26,33,34,35,36]. Undetectable in undifferentiated and early differentiating embryonic stem cells, miR-99a expression abruptly increases in day six of cardiac differentiation to regulate the expression of mesodermal cardiac genes Mesp1, Nkx2.5 and Mef2c and of TGF-beta signaling, presumably through changes in the activity of ATP-dependent chromatin remodeling complexes [26]. Should miR-99a expression signal activation of cardiomyogenesis mechanisms in vivo, its overexpression in (female) DS hearts might represent a precocious activation of cardiomyogenesis (which has been shown to lead to cardiac defects in mice) and explain the prevalence of cardiac defects in DS female individuals [25,26,37,38].

Congenital heart diseases (CHD) are among the most common malformations in children, with a complex, not fully understood ethiopathology that also involves microRNAs (Dicer conditional knockout leads to significant chamber septation defects) [39,40,41]. Of note, up to 60% of the DS newborns show various types of CHDs, a phenotype associating deregulation of several signaling pathways, including VEGF-A, ciliome, hedgehog, folate, ECM-receptor, purine metabolism, cell cycle and Wnt signaling [29,42,43,44,45]. Most of the studies analyzing gender-stratified data related to CHD in DS have shown a surprising (and, to the best of our knowledge, unexplained) prevalence of CHD in DS females. [29,30,46,47,48,49,50,51,52,53]. Although still mechanistically not fully understood, cardiac overexpression of miR(21) in DS hearts might play a distinct post-transcription regulatory role, part of the overall picture of imbalanced microRNA expression contributing to gene dosage effects in DS individuals [25,54].

Our KEGG pathway analysis of the miR99a miRWalk-predicted targets expressed in DS amniocytes and fetal hearts using the GATHER platform points towards two conserved signaling pathways: focal adhesion and cytokine–cytokine receptor interactions.

Focal adhesion (FA) signaling has an evolutionary conserved role in the septation of the vertebrate heart and has been previously suggested to be deregulated in fetal DS hearts [55,56,57,58,59]. However, the role of microRNAs in deregulation of FA in the DS heart has not been explored so far. Of note, focal adhesion signaling has also been found to be deregulated in the peripheral blood mononuclear cell (PBMCs) of DS neonates and children, indicating a wider, non-morphogenetic role of FA in DS pathogenesis [60]. It is worth mentioning that the DS-associated cell adhesion molecules DSCAM and Col6A2, highly expressed during vertebrate heart development and strong candidates for a role in DS-related CHDs, are also predicted by miRWalk 3.0 algorithm to be targeted by miR-99 [61,62].

To the best of our knowledge, the involvement of cytokine–cytokine receptor interactions in the pathogenesis of DS-related CHDs has not been described so far. Aside the systemic increase in serum pro-inflamatory cytokines, an increase in the myocardial synthesis of pro-inflammatory cytokines (IL-1b, IL-6, TNF-α), but also of IL-10 (predicted to be targeted by miR-99a), has been reported in euploid children with congenital cardiac defects, presumably an effect of pressure overload and correlate with serum levels [63]. Although most of the data point towards a hemodynamic overload of the DS hearts with septal defects as the cause of these changes, recent research has identified two major signaling hubs, JNK and NF-kB able to transduce cytokine signaling in the vertebrate myocardium and having a significant ethiopathogenic potential in children with congenital heart defects [64]. Experimental alteration of NF-kB in the cardiac primordium significantly alters the development of the outflow tract (the transient embryonic structure that connects the aortic sac with the ventricles and whose dismorphogenesis leads to conotruncal congenital heart defects) [65,66]. JNK is a rather promiscuous integrator of several signaling pathways, of which several have been shown to be linked to cardiac defects, including ERK1/2 and Wnt/non-canonical signaling [67,68]. Of note, both JNK1 and NF-kB are among the miRWalk3.0 targets predicted to interact with miR-99a with binding probabilities over 90%.

## 5. Conclusions

Our data strongly suggest the increase of AF miR-99a levels in female DS samples is not a transcriptional event, but most probably signals a lower rate of decay of the mature form of the microRNA. microRNA decay is far less understood than microRNA biogenesis, and multiple factors (from genomic organization, structural heterogeneity and post-transcriptional modifications to target abundance and biological context) are known to modulate miRNA degradation [69]. To the best of our knowledge, this is the first report pointing towards an altered stability/decay of a microRNA in the trisomic milieu, which offers a new molecular mechanism for understanding gene dosage in Down syndrome.

Despite our lack of data regarding the cardiac anomalies in the DS pregnancies included in our study, we dare to propose a possible novel association of AF miR-99a levels and cardiac defects in female-fetus pregnancies in general and DS in particular. Further studies, on a much wider lot of samples, are needed to understand whether the augmented levels of miR-99a in the amniotic fluid are predictive for fetal cardiac defects and whether the suggested alterations of post-transcriptional processing/decaying events are also present in male DS fetuses.

## Figures and Tables

**Table 1:** Clinical characteristics of the patients.

		DS (*n* = 17) (Mean ± SD)	E (*n* = 22) (Mean ± SD)	*p* Value (*t*, df)
Maternal age	M + F	35.65 ± 5.11	34.98 ± 4.98	0.684 (0.4105, 34.09)
	M	36.83 ± 5.31	34.88 ± 6.17	0.537 (0.6367, 11.70)
	F	35.00 ± 5.14	35.04 ± 4.41	0.986 (0.0183, 19.83)
Gestational age	M + F	17.17 ± 1.85	18.44 ± 1.72	0.036 (2.184, 33,3)
	M	17.13 ± 2.42	17.96 ± 1.21	0.467 (0.77, 6.89)
	F	17.19 ± 1.59	18.71 ± 1.95	0.043 (2.14, 22.94)
% Males		35.29	36.36	0.944 *

Heteroscedastic, two-tailed Student *T* test, Welch’s correction; * *Z*-test; DS—Down syndrome samples; E—Euploid samples; M—Males; F—Females.

**Table 2:** MicroRNA fold changes in Down syndrome vs. euploid amniotic fluid samples.

	Fold Changes (*p* Value/*t*, df)		
	M + F	M	F
let7c	0.61 (0.422/0.82, 23.49)	0.12 (0.239/1.29, 6.69)	1.16 (0.612/0.52, 20.37)
miR-125b-2	1.04 (0.998/0.002, 35.95)	0.51 (0.280/1.14, 10.66)	1.35 (0.357/0.94, 22)
miR-155	1.16 (0.504 *)	0.81 (0.282 *)	2.08 (0.072 *)
miR-99a	2.07 (0.166/1.42, 26)	0.61 (0.173/1.52, 7.08)	3.84 (0.0005/4.13, 21.64)
Pri-miR-99a	0.58 (0.731/0.34, 33.77)	0.35 (0.7127/0.38, 11.04)	0.8 (0.889/0.14, 17.6)

Heteroscedastic, two-tailed Student *T* test, Welch’s correction; * two-tailed Mann–Whitney test; M—Males; F—Females.

**Table 3:** MicroRNA fold changes (males vs. females) in Down syndrome vs. euploid amniotic fluid samples.

Males vs. Females	Euploid	DS
	Fold Change (*p* Value/*t*, df)	Fold Change (*p* Value/*t*, df)
miR-99a	2.12 0.053 (2.093, 16.05)	5.12 0.040 (2.541, 6.702)
let7c	1.06 0.876 (0.1594, 11.22)	0.29 0.199 (1.453, 5.752)
miR-125b2	1.40 0.361 (0.9408, 15,82)	0.65 0.281 (1.157; 7.848)
miR-155	0.20 (0.6452 *)	0.08 (0.0031 *)
primiR-99a	1.00 0.998 (0.001839, 17.28)	1.15 0.759 (0.3164, 7.901)

Heteroscedastic, two-tailed Student *T* test, Welch’s correction; * two-tailed Mann–Whitney test.

**Table 4:** Network-inferred KEGG pathway analysis of predicted miR-99a targets in Down syndrome-amniocytes and Down syndrome fetal hearts.

	Amniocytes (Bayes Factor)	Fetal Heart (Bayes Factor)
1	mmu04510:Focal adhesion (19.41)	hsa04060:Cytokine–cytokine receptor interaction (21.21)
2	mmu04060:Cytokine–cytokine receptor interaction (15.71)	mmu04510:Focal adhesion (14.84)
3	rno04010:MAPK signaling pathway (14.13)	mmu04060:Cytokine–cytokine receptor interaction (14.77)
4	mmu04010:MAPK signaling pathway (12.92)	hsa04630:Jak-STAT signaling pathway (11)
5	hsa04060:Cytokine–cytokine receptor interaction (12.28)	mmu05010:Alzheimer’s disease (10.89)
6	mmu04210:Apoptosis (10.07)	rno04060:Cytokine–cytokine receptor interaction (10.41)

## Supplementary Materials

The following are available online at https://www.mdpi.com/1010-660X/55/11/728/s1, Table S1: List of amniocyte and fetal heart genes data set (gene symbols) predicted to be targeted by miR-99a.
