# Supplementary material for: Mature miR-99a Upregulation in the Amniotic Fluid Samples from Female Fetus Down Syndrome Pregnancies: A Pilot Study
Source: Medicina (Kaunas). 2019 Nov 7;55(11):728. doi: 10.3390/medicina55110728 (PMC6915350; doi:10.3390/medicina55110728)
Supplement: Supplementary file 1 [file medicina-55-00728-s001.pdf]

Table S1. List of amniocytes and fetal heart genes data set (gene symbols) predicted to be targeted by miR-99a.

**Target genes amniocytes (GSE16176)**		**Target genes fetal heart (GSE1789, GSE1397)**	
ZNF770	MLXIP	ZSWIM8	DNAH9
ZNF687	MEF2C	ZNF667	DDX23
ZNF652	MAPK4	ZNF557	CYP4B1
ZNF585A	MAATS1	ZNF3	CYLD
ZNF418	LSAMP	ZBTB20	CSF2RB
ZNF3	LMO3	WASL	CPNE6
ZMYND8	LDLRAD4	VDR	COMMD4
ZKSCAN7	LATS2	UPK1B	CLCN4
ZKSCAN3	KYNU	UCP3	CENPE
ZFYVE1	KLHL42	TRIO	CDADC1
ZBTB39	KIAA1644	TRIM9	CD72
YTHDF3	KANK2	TRIM31	CD163
XIAP	ITGB3	TRIM23	CCNT2
WNT10B	ISG20L2	TPD52	CAPN10
WHRN	IGIP	TM6SF1	CABP5
WDR90	HNRNPU	TGFB2	CA6
VPS26B	HEPH	TF	C1orf56
VCAN	H2AFY	TECPR2	C1orf109
VASH2	GPR161	TBL1X	ASB12
UBE2G2	GPATCH4	SYT2	ARHGAP26
TTLL4	GOLGB1	SYK	AP1M2
TTC26	GGNBP2	SUGP2	ANKRD12
TRPV2	GGACT	STYK1	AKAP13
TRIO	FZD3	STXBP2	ACSM3
TRIM3	FXR2	STS	ACSL3
TRAF3IP3	FST	SNCAIP	ACOX1
TP63	FOXO3	SMARCD2	ABCB11
TNS4	FOXN3	SLC2A4	ABCB1
TNRC18	FLNA	SLC1A5	ZNF423
TNK2	FHAD1	SIGLEC5	ZNF407
TNFRSF1A	FGD6	SFPQ	ZNF175
TNFAIP8L1	FANCA	SEC23IP	XRCC6
TNFAIP2	FAM76A	SCN11A	VCAN
TGFB2	FAM153A	SCAI	VAV3
TBC1D9	FAM110B	RXRA	USP12
TAF3	EYA3	RSRC1	TSEN2
SYNPO2	EXOSC2	RNASE6	TRIM58
SYNE1	ELP2	RERE	TRIM29
STXBP5L	ELMO1	RASGRF1	TRDN
STXBP4	EGF	PYCRL	TP63
STAMBPL1	DYNC2LI1	PRX	TMCC1
SRSF3	DST	PRPF19	TCF7L2
SPTBN1	DPPA4	PRKAA1	TBC1D3
SMURF2	DNLZ	PRICKLE3	TAF6L
SLTM	DNAH5	PPP6R2	SULT2A1
SLFNL1	DLGAP4	PPIG	SPTA1
SLC6A2	DLG1	POU3F2	SLC6A2
SLC45A4	DIAPH2	POLR3G	SLC4A1
SLC35E2	DDX3X	PLXNA2	SLC35D1
SLC22A13	DAPK1	PLEKHO1	SHOX2
SIK3	DAAM2	PKDREJ	SECISBP2L
SIGLEC5	CTNND1	PIP5K1A	RWDD2B
SHOX2	CTNNA1	PIK3R4	RELN

SH3TC2	CSNK1A1	PIGH	RAB27A
SGPP2	CSGALNACT2	PFKFB4	PRRC2C
SGCD	CRB1	PDE4DIP	PRMT2
ROMO1	CNOT4	PDCD2	PRKCQ
RNF41	CLIC5	PDAP1	PLCB4
RNF141	CLDN2	PASK	PHTF1
RFX3	CKAP5	PADI2	PDK3
RERE	CFLAR	P2RY10	PDGFRL
RCBTB1	CEP192	OSR2	PAPOLA
RBM6	CECR1	ORC4	NUP160
PTGS1	CD93	OLFM1	NQO1
PSMF1	CD300LG	NF2	NOX3
PRICKLE3	CCDC63	MYO7A	NOL3
PPP2R2A	CCDC149	MTERF1	NGFR
PPARGC1A	CAMTA2	MSLN	NF1
PPARA	C20orf194	MSH4	MLC1
POPDC2	C16orf45	MPZL2	METTL16
POLR2A	C10orf55	MASP2	MED20
PLCG2	BRI3BP	MASP1	LDLRAD4
PDZD8	BNC2	MAPK4	KPNB1
PARP14	BCOR	LMO3	KCTD12
PADI1	BCAT1	LHX2	KCNN4
OR8B2	BBX	LDAH	KCNJ9
OLFM1	BACE2	KMT2A	ITPR2
NRBF2	B4GALNT4	KITLG	ITGB3
NOL3	ATP8B3	KIAA1456	IFNAR1
NMNAT2	ARID2	IL10	GPR161
NIPBL	ARHGEF12	IDS	GLRA3
NF2	AQP4	ICMT	GAS2
NDOR1	AP1M2	ICAM3	FECH
NCOA1	AGRP	HOXB3	ERG
NBEAL2	ADGRL1	HOXA3	ENTPD7
MYCBP2	ACSM2B	H6PD	ENTPD1
MSTO1	ACER3	GUCA1A	ENOSF1
MR1	ABLIM2	GRM7	DOC2A
	ABCC1	GREM1	DDX3X
		GNAO1	CTNNA1
		GLB1L2	CNOT4
		GAS7	CLGN
		GALNT4	CHTOP
		FZD9	CDH11
		FOXI1	CCT8
		FGF16	CASK
		FCAR	C5
		FAM168A	BDH1
		EXPH5	BACE2
		EXOSC2	ARID3A
		EPHA3	ARHGEF10
		EPHA1	AQP4
		E4F1	APP
		E2F3	ANKS1B
		DYNC2LI1	ANGPT1
		DYNC1I1	ALPK3
		DSCAM	ADGB
		DNAH9	ADAMTS7
			ACKR2
			ABL2
